# Selinexor Enhances NK Cell Activation Against Malignant B Cells *via* Downregulation of HLA-E

**DOI:** 10.3389/fonc.2021.785635

**Published:** 2021-12-01

**Authors:** Jack G. Fisher, Christopher J. Walker, Amber DP. Doyle, Peter WM. Johnson, Francesco Forconi, Mark S. Cragg, Yosef Landesman, Salim. I. Khakoo, Matthew D. Blunt

**Affiliations:** ^1^ School of Clinical and Experimental Sciences, University of Southampton, Southampton, United Kingdom; ^2^ Research & Translational Development, Karyopharm Therapeutics, Newton, MA, United States; ^3^ School of Cancer Sciences, University of Southampton, Southampton, United Kingdom

**Keywords:** NK cells, natural killer cells, selinexor, NKG2A, HLA-E, XPO1, lymphoma, CLL (chronic lymphocytic leukemia)

## Abstract

Selinexor is an FDA approved selective inhibitor of the nuclear export protein exportin-1 (XPO1) and causes specific cancer cell death *via* nuclear accumulation of tumor suppressor proteins. Design of rational studies for the use of selinexor in combination with other therapeutic agents, such as immunotherapies, requires a fundamental understanding of the effects of selinexor on the immune system. One important emerging area of immunotherapy are natural killer (NK) cell based therapeutics. NK cell function is tightly regulated by a balance of signals derived from multiple activating and inhibitory receptors. Thus in cancer, up-regulation of stress ligands recognised by activating receptors or down-regulation of HLA class I recognised by inhibitory receptors can result in an anti-cancer NK cell response. Changes in XPO1 function therefore have the potential to affect NK cell function through shifting this balance. We therefore sought to investigate how selinexor may affect NK cell function. Selinexor pre-treatment of lymphoma cells significantly increased NK cell mediated cytotoxicity against SU-DHL-4, JeKo-1 and Ramos cells, concurrent with increased CD107a and IFNγ expression on NK cells. In addition, selinexor enhanced ADCC against lymphoma cells coated with the anti-CD20 antibodies rituximab and obinutuzumab. In probing the likely mechanism, we identified that XPO1 inhibition significantly reduced the surface expression of HLA-E on lymphoma cell lines and on primary chronic lymphocytic leukemia cells. HLA-E binds the inhibitory receptor NKG2A and in accordance with this, selinexor selectively increased activation of NKG2A+ NK cells. Our data reveals that selinexor, in addition to its direct cytotoxic activity, also activates an anti-cancer immune response *via* disruption of the inhibitory NKG2A:HLA-E axis.

## Introduction

Natural killer (NK) cells are innate immune effectors which induce direct cytotoxicity against tumor cells and mediate antibody dependent cellular cytotoxicity (ADCC). Deficiency of NK cell function and number is associated with increased development of cancer (1). The infiltration of NK cells within tumors is associated with improved outcome for a number of cancers (2) whilst NK cells are also associated with survival during anti-PD-1 antibody therapy (3, 4). In patients with non-Hodgkin lymphoma receiving anti-CD20 based chemoimmunotherapy, low number of NK cells is associated with shorter progression free survival (5). In addition to their direct cytolytic function, NK cells promote optimal CD8+ T cell responses *via* release of tumor antigens, recruitment and maturation of dendritic cells, as well as IFNγ mediated upregulation of MHC I expression (6, 7). Furthermore, IFNγ production by NK cells has also recently been shown to sustain dormancy of liver metastases (8).

In contrast to T and B lymphocytes, NK cell activation against malignant cells is tightly controlled *via* the integration of signals from an array of germline encoded, non-rearranged, surface receptors (2, 7, 9, 10). Downregulation of HLA molecules on target cells is detected by the inhibitory killer cell immunoglobulin-like receptor (KIR) family and NKG2A, which specifically detects HLA-E, leading to loss of inhibition and NK cell activation. In contrast, upregulation of cell stress associated ligands is detected by a variety of activating receptors expressed by NK cells including NKp30, NKp44, NKp46 and NKG2D. In addition, NK cells express other activating receptors including CD16, NKG2C as well as activating KIRs. Due to their potent anti-tumor functions, the enhancement of NK cell activity against cancer is currently the focus of multiple therapeutic strategies. These include CAR-NK based approaches (11, 12), agonistic antibodies (13), cytokine mediated stimulation (14) and checkpoint inhibitors (15). In addition to these direct approaches, NK cells can also contribute to the efficacy of other cancer therapies *via* the detection of altered activating and inhibitory ligand expression patterns on stressed tumor cells. For example, NK cell activation has been reported to be enhanced following tumor exposure to cytostatic drug combinations (16), proteasome inhibitors (17), genotoxic agents (18) and ionizing radiation (19).

Exportin-1 (XPO1) is a nuclear export protein which transports cargo proteins with a leucine-rich nuclear export signal (NES) and ribosomal subunits from the nucleus to the cytoplasm (20). This activity ensures that the correct cellular location of proteins is achieved and is crucial for normal cell translational activity and function (21). Upregulation of XPO1 is common in human cancers and results in abnormal tumor suppressor protein export with imbalance favoring proto-oncogene activity. Increased XPO1 expression is negatively associated with survival in various cancers including diffuse large B cell lymphoma (DLBCL) (22, 23) and mantle cell lymphoma (24). Targeted inhibition of XPO1 by the selective inhibitor selinexor leads to cancer cell death through accumulation of tumor suppressor proteins in the nucleus, dysregulation of growth regulatory proteins and blockade of oncogene protein translation (21). In addition, selinexor causes degradation of XPO1 protein in a proteasome dependent mechanism (25). The therapeutic efficacy of XPO1 inhibition in patients has led to FDA approval of selinexor for the treatment of patients with multiple myeloma and DLBCL in the USA, and conditional marketing authorization by the European Commission for patients with multiple myeloma. Various clinical trials are also ongoing to assess selinexor for the treatment of solid tumors and hematological malignancies (20), including in combination with anti-CD20 antibodies for patients with advanced B cell non-Hodgkin lymphoma (NCT03147885) (26). In addition to its direct cytotoxicity against tumor cells, selinexor has also been described to sensitize breast cancer cells to T cell attack in combination with a TRAIL-R2xCD3 bispecific antibody (27) and to increase CAR T cell activity against CD19 positive malignant B cells (28). The effect of selinexor or XPO1 inhibition on cancer cell sensitivity to NK cell activity however has not previously been investigated.

In this study, we evaluated the effect of XPO1 inhibition on human NK cell activation against lymphoma cells. Our data identifies that XPO1 inhibition sensitizes lymphoma cell lines to NK cell mediated killing *via* downregulation of HLA-E and subsequent activation of NKG2A+ NK cells. This study therefore reveals that selinexor, in addition to its direct cytotoxic activity, also triggers an innate immune response *via* disruption of the inhibitory NKG2A:HLA-E axis.

## Materials and Methods

### Reagents and Cell Lines

SUDHL4 (ATCC, CRL-2957), JeKo-1 (ATCC, CRL-3006) and RAMOS (ATCC, CRL-1596) cells were cultured in R10 medium (RPMI 1640 [Gibco] with 1% penicillin-streptomycin [Life Technologies] and 10% heat inactivated fetal bovine serum [FBS; Sigma]). Cells were treated with 50, 500 or 2000nM selinexor (KPT-330, provided by Karyopharm Therapeutics), 50nM leptomycin B (Sigma) or DMSO control (for the indicated 0nM negative controls) for 16 hours at 37°C before use. To prevent drug-induced apoptosis, cell lines were incubated with Q-VD-OPh (QVD) (10-20µM) (Sigma) for 30 minutes prior to addition of XPO1 inhibitors or DMSO control.

### Peripheral Blood Mononuclear Cell (PBMC) Isolation and NK Cell Purification

Healthy donor peripheral blood mononuclear cells (PBMC) were obtained with full ethical approval from the National Research Ethics Committee (reference 06/Q1701/120). PBMC from patients with chronic lymphocytic leukemia (CLL) were collected from patients attending clinic at Southampton General Hospital. All patients provided written informed consent and the study was approved by the Institutional Review Boards at the University of Southampton (REC: H228/02/t). PBMCs were cryopreserved and stored in liquid nitrogen. CD56+CD3- NK cells were isolated from cryopreserved healthy PBMCs using the Miltenyi human NK cell isolation kit and cultured in R10 medium at a density of 1.5x10^6^ cells/mL and incubated with 1 ng/mL IL-15 (R&D Systems) overnight before use in functional assays.

### NK Cell Cytotoxicity Assay

B cell lymphoma cell lines were stained with Cell Trace™ Violet Cell Proliferation Kit (Invitrogen™) following the manufacturer’s instructions then incubated with QVD and selinexor or DMSO control for 16 hours. Isolated NK cells were then co-cultured with lymphoma cells at an effector: target (E:T) ratio of 5:1 for 4 hours at 37°C. After co-culture, cells were stained with 1.6 µg/mL propidium iodide (Invitrogen™) and NK cell specific lysis of Violet stained target cells assessed by flow cytometry. Cells were acquired on a BD FACS Aria II (BD Biosciences) machine using FACSDiva software (BD Biosciences) and analysed with FlowJo v10.7.1 (BD Biosciences). Lysis was defined as uptake of propidium iodide by the target cells.

### Assessment of NK Cell Degranulation and Cytokine Production

PBMCs were incubated with 1 ng/mL IL-15 overnight at a cell density of 2x10^6^ cells/mL and then co-cultured with selinexor- or leptomycin B-treated lymphoma cell lines at an E:T ratio of 5:1 for 4 hours at 37°C. Immediately before co-culture, 0.17 µg/mL α-CD107a (LAMP)-eFluor660 (clone eBioH4A3, Invitrogen) was added to PBMCs. After 1-hour of co-culture, GolgiStop (per manufacturer recommendations, BD Biosciences) was added. Following 4-hours of incubation, cells were incubated with 10% human serum at 4°C for 15 minutes before surface staining with antibodies against CD3-PerCP (clone UCHT1, Biolegend), CD56-PE/Cy7 (HCD56, Biolegend) and NKG2A-FITC (REA110, Miltenyi Biotech) in FACS buffer (PBS, BSA 1%, Sodium Azide 0.05%) at 4°C for 30 minutes. Cells were then permeabilized and fixed with BD Cytofix/Cytoperm (BD Biosciences) per manufacturer recommendations and stained with anti-IFNγ-BV421 (BD Biosciences) at 4°C for 30 minutes. Cells were then washed twice with 1X Perm/Wash buffer and immediately assessed by flow cytometry using a BD FACS Aria II (BD Biosciences) and FACSDiva software (BD Biosciences) as above.

### Assessment of NK Cell Ligand Expression on Lymphoma Cells, Primary CLL Cells and Normal Lymphocytes

Ramos, SU-DHL-4 and JeKo-1 cells were incubated with selinexor (50-2000nM), leptomycin B (50nM) or DMSO control for 16 hours in the presence of QVD. Cells were then surface stained with antibodies against activating NK cell ligands Vimentin-A488 (clone 280618, R&D Systems), ULBP-1-PE (170818, R&D Systems), ULBP-2/5/6-PerCP (165903, R&D Systems), CD54-PB (HCD54, Biolegend), B7H6-APC (875001, R&D Systems) and MICA/B-PE/Cy7 (6D4, Biolegend); the inhibitory NK cell ligand HLA-E-PE/Cy7 (3D12, Biolegend) and pan-HLA class-I molecules (W6/32, Biolegend) for 30 min at 4°C. CLL cells were incubated with selinexor (50-2000nM) or DMSO control for 40 hours in the presence of QVD then incubated with 10% human serum at 4°C for 15 minutes before CD5+CD19+ CLL cells were surface stained with anti-HLA-E-PE/Cy7 (3D12, Biolegend) for 30 min at 4°C. Normal PBMC were incubated with selinexor (500-2000nM) or DMSO control for 16 hours then surface stained with anti-HLA-E-APC (3D12, Biolegend), anti-CD3-PerCP (UCHT1, Biolegend), anti-CD19-PE (HIB19, Biolegend) and anti-CD56-PE/Cy7 (HCD56, Biolegend) for 30 min at 4°C. Cells were then acquired on a BD FACS Aria II (BD Biosciences) using FACSDiva software (BD Biosciences) as above.

### Immunoblotting

Ramos, SU-DHL-4 and JeKo-1 cells were incubated with selinexor (50-2000nM) or leptomycin B (50nM) for 16 hours in the presence of QVD then lysed in NP40 Cell Lysis Buffer (Fisher Scientific UK) supplemented with PMSF (Sigma, 174 µg/mL) and protease inhibitor (Sigma, 1:100 final dilution). Proteins were separated on 10% polyacrylamide gels (Thermo Fisher Scientific), transferred to nitrocellulose membranes (Amersham) and blocked in 5% BSA (Sigma) before being probed with antibodies against XPO1 (D6V7N, Cell Signalling Technology), PARP (4C10-5, BD Pharmingen), HLA-E (Sigma), p53 (1C12, Cell Signalling Technology) or β-actin (8H10D10, Cell Signalling Technology). Protein bands were detected following incubation with HRP-linked secondary antibodies (Dako) and chemiluminescence reagents (Thermo Scientific) and were visualized using the ChemiDoc-It imaging system (UVP). Primary and secondary antibodies were used at concentrations recommended by the manufacturer.

### Assessing the Impact of Selinexor on ADCC

SU-DHL-4 cells were incubated with selinexor (50-2000nM) for 16 hours in the presence of QVD then incubated with the anti-CD20 antibodies rituximab or obinutuzumab or isotype control (1 µg/mL) for a further 20 minutes at 37°C prior to co-culture with PBMC (degranulation assay) or isolated NK cells (cytotoxicity assay) as described above.

### Statistical Analysis

Statistical analyses were performed using GraphPad Prism V.9.0 software. Paired t-test was used to compare differences between the means of two groups and paired or unpaired one-way ANOVA followed by Dunnett’s *post-hoc* test analysis was used to compare differences in means between multiple groups. Significance values are defined as: *P < 0.05, **P < 0.01, ***P < 0.005 and ****P < 0.001.

## Results

### Selinexor Enhances NK Cell Cytotoxicity Against Lymphoma Cell Lines

**Figure 1:**
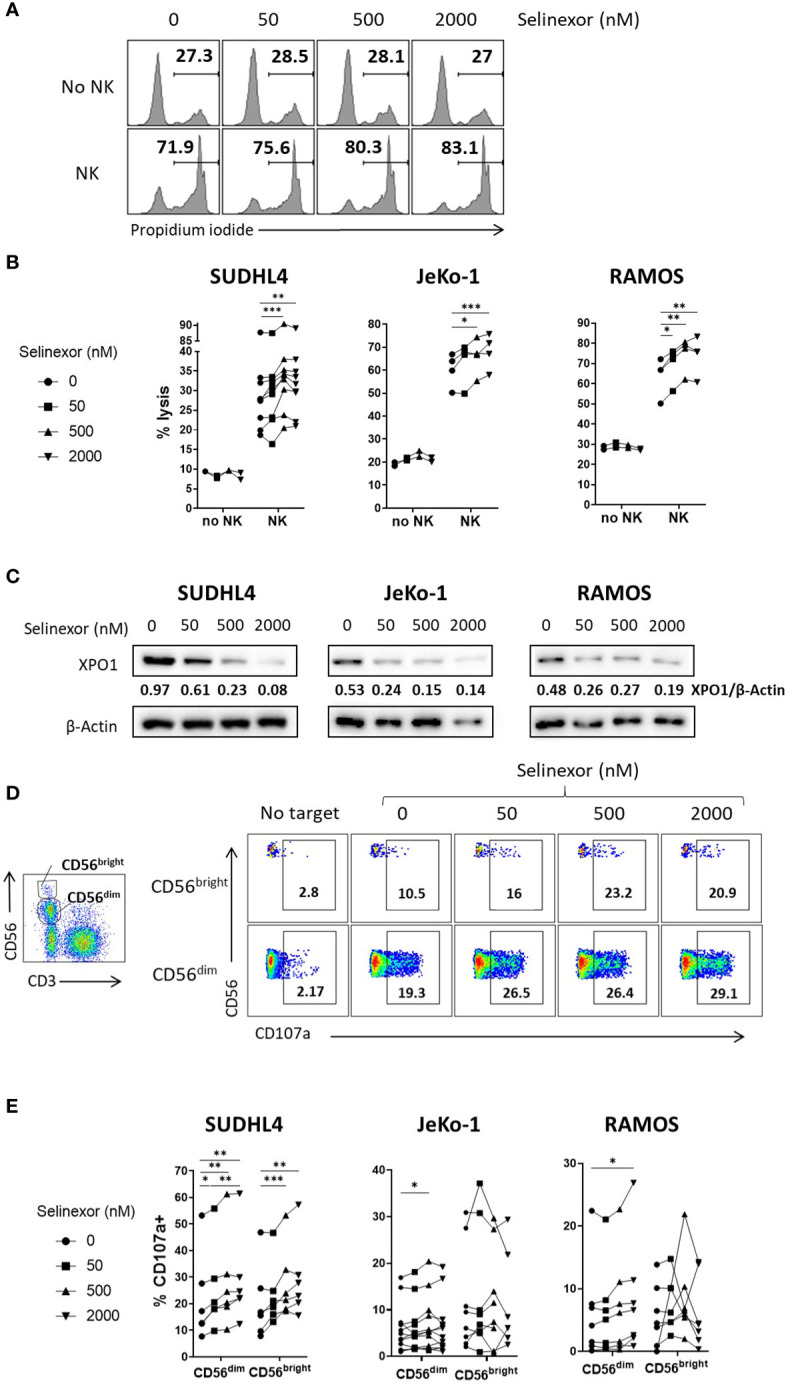
Selinexor increases NK cell cytotoxicity against lymphoma cells. **(A, B)** Isolated NK cells incubated with IL-15 overnight were co-cultured for 4 hours with SUDHL4, n=9; JeKo-1, n=4; or RAMOS, n=4 cells (E:T (effector:target) ratio 5:1) that were pre-treated for 16hrs with selinexor at indicated concentrations or DMSO control in the presence of the caspase inhibitor QVD. Cytotoxicity was then assessed using propidium iodide staining on violet dye stained target cells. Displayed is a representative example for RAMOS target cells in A) and data as % of propidium iodide+ target cells in B). N numbers indicate independent NK cell donors. Data was analysed with paired one-way ANOVA followed by Dunnett’s *post-hoc* test analysis: *P < 0.05; **P < 0.01; ***P < 0.005. **(C)** SUDHL4, JeKo-1, and RAMOS cells were incubated for 16hrs with selinexor at indicated concentrations or DMSO control in the presence of the caspase inhibitor QVD. XPO1 and β-actin protein levels were then detected by immunoblotting. Representative of two independent experiments. **(D, E)** Healthy human PBMCs were incubated with IL-15 overnight then co-cultured (E:T 5:1) for 4hrs with SUDHL4 (n=6), JeKo-1 (n=12) and RAMOS (n=7) cells pre-treated with selinexor at indicated concentrations or DMSO control for 16hrs. Degranulation (CD107a) was then assessed on CD56^dim^ and CD56^bright^ NK cells identified as indicated in the representative example. Representative example of enhanced NK cell degranulation after co-culture of PBMCs with selinexor-treated SUDHL4 is shown in **(D)**. CD107a normalized to the ‘no target’ control is shown in **(E)**. Groups were analysed with repeated measure one-way ANOVA followed by Dunnett’s *post-hoc* test analysis: *P < 0.05; **P < 0.01; ***P < 0.005.

To assess whether selinexor modulated NK cell activation against malignant B cells, we incubated selinexor (50-2000 nM) for 16 hours with SUDHL4 (DLBCL), JeKo-1 (mantle cell lymphoma) or RAMOS (Burkitt lymphoma) cells and then assessed cytotoxicity following co-culture with NK cells isolated from healthy human donors. Selinexor increased NK cell cytotoxicity in a concentration-dependent manner against SUDHL4 (p<0.005), JeKo-1 (p<0.005) and RAMOS (p<0.01) cells (**Figures 1A, B**). This lysis was specific to NK cell activity because in these experiments apoptosis induced by selinexor was prevented by addition of the caspase inhibitor QVD, as shown by no increase in propidium iodide staining (**Figures 1A, B**) in the absence of NK cells. These results indicate that blockade of intrinsic apoptosis in lymphoma cells, and hence resistance to direct selinexor toxicity, does not prevent NK mediated lysis stimulated by selinexor. This is in agreement with previous reports of caspase independent cell death induced by effector lymphocytes and purified granzyme B (29, 30). Selinexor activity was confirmed by clear concentration-dependent XPO1 degradation (**Figure 1C**), as previously reported (31). In accordance with our observations that selinexor enhanced NK cell activity against lymphoma cells, we measured degranulation (CD107a) of CD56^dim^ and CD56^bright^ NK cell subsets when co-cultured with these target cells. This revealed that selinexor significantly increased the degranulation of the more mature and cytotoxic CD56^dim^ NK cell population against all cell lines tested compared to untreated controls (**Figures 1D, E**). Degranulation of the more regulatory and proliferative CD56^bright^ subgroup of NK cells was significantly enhanced by selinexor against SUDHL4 cells (**Figure 1E**). Together these data demonstrate that the XPO1 inhibitor selinexor increases NK cell cytotoxicity against B lymphoma cell lines.

### Selinexor Downregulates Surface HLA-E Expression on Tumor Cells and Selectively Activates NKG2A+ NK Cells

**Figure 2:**
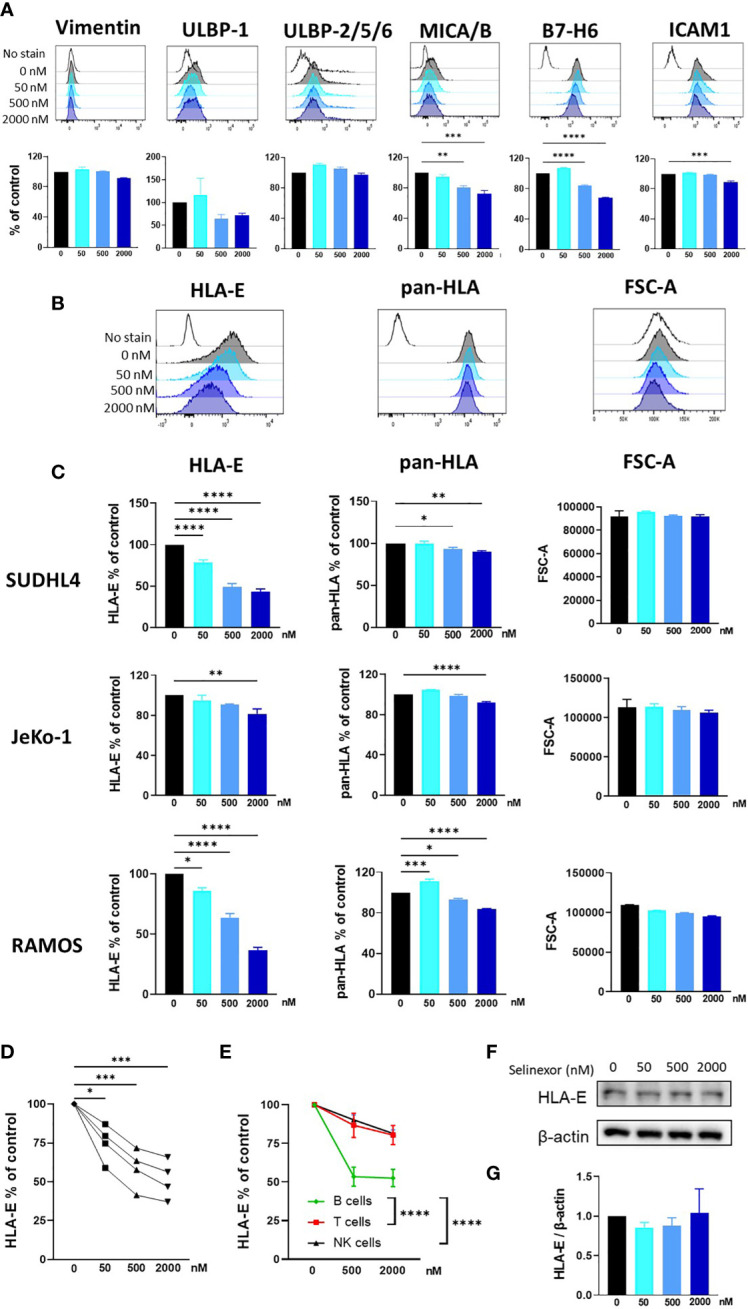
Selinexor decreases the surface expression of HLA-E on malignant B cells. **(A)** SUDHL4 cells were incubated for 16hrs with selinexor at indicated concentrations or DMSO control in the presence of the caspase inhibitor QVD then assessed for surface expression of Vimentin, ULBP-1, ULBP-2/5/6, MICA/B, B7-H6 and ICAM1 by flow cytometry. Representative data and summarized data as % of control is shown from three independent experiments. **(B, C)** SUDHL4, JeKo-1, and RAMOS cells were incubated for 16hrs with selinexor at indicated concentrations or DMSO control in the presence of the caspase inhibitor QVD then assessed for surface expression of HLA-E (clone 3D12), class-I HLA proteins (pan-HLA, clone W6/32) and cell size as measured by the FSC-A parameter. Representative histograms of SUDHL4 cells are shown in **(B)** and summarized data mean ± SEM of SUDHL4 (n=8), JeKo-1 (n=5) and RAMOS (n=3) cells are shown in **(C)**. Data was analysed using one-way ANOVA followed by Dunnett’s *post-hoc* test analysis: *P < 0.05, **P < 0.01, ***P < 0.005, ****P < 0.0001. **(D)** Primary CLL cells were incubated for 40hrs with selinexor at indicated concentrations or DMSO control in the presence of the caspase inhibitor QVD then CD5+CD19+ CLL cells were assessed for surface expression of HLA-E (clone 3D12). Shown is HLA-E % of DMSO control from four different CLL patient samples. Data was analysed using one-way ANOVA followed by Dunnett’s *post-hoc* test analysis: *P < 0.05, ***P < 0.005. **(E)** Healthy human PBMC were incubated with selinexor (500-2000nM) or DMSO control for 16hrs then assessed for surface expression of HLA-E (clone 3D12) on B cell, T cell and NK cell populations. Summarized data as HLA-E % of control is shown from six different donors. Data was analysed using two-way ANOVA: ****P < 0.0001. **(F, G)** SUDHL4 cells were incubated for 16hrs with selinexor at indicated concentrations or DMSO control in the presence of the caspase inhibitor QVD. HLA-E and β-actin protein levels were then detected by immunoblotting. Representative images are shown in **(F)** and summarized data mean ± SD protein band intensity relative to β-actin (n=2) is shown in **(G)**.

NK cell activity is tightly controlled by a plethora of activating and inhibitory receptors which recognize and engage ligands expressed on the surface membrane of infected, transformed or stressed target cells (7, 10). Therefore, to investigate the mechanism for enhanced NK cell activation and killing following selinexor incubation with lymphoma cells, we screened SUDHL4, JeKo-1 and RAMOS cells by flow cytometry for changes in a panel of ligands for activating and inhibitory NK cell receptors. Following selinexor incubation for 16 hours (50-2000nM), no significant change was evident in expression of the activating ligands Vimentin and ULBP-2/5/6, a trend to down-regulation for ULBP-1 and a significant reduction in expression of MICA/B, B7-H6 and ICAM1 (**Figure 2A**). Signals from these activating receptors do not account for the increased NK cell activation noted following selinexor pre-treatment of lymphoma cells (**Figure 1**). Therefore we assessed expression of ligands for inhibitory NK cell receptors. The surface expression of HLA-E as determined by the HLA-E specific antibody 3D12 was downregulated by selinexor on SUDHL4 (57% reduction, p<0.001), JeKo-1 (19% reduction, p<0.01) and RAMOS (63% reduction, p<0.001) cells in a concentration-dependent manner (**Figures 2B, C**). This was not due to a decrease in cell size as there was no change in forward scatter following selinexor treatment (**Figures 2B, C**). In addition, selinexor caused a much lower, but statistically significant, downregulation of total HLA molecules as measured by the antibody clone W6/32 (**Figures 2B, C**). This decrease likely corresponds to the downregulation of HLA-E as the W6/32 antibody clone recognizes the HLA proteins HLA-A, -B, -C in addition to HLA-E (32, 33). Consistent with this model, HLA-E represents a relatively small fraction of total HLA on the surface of cell. We then assessed whether selinexor modulated the expression of HLA-E on primary tumor cells using samples derived from patients with CLL. Selinexor reduced surface HLA-E expression on CD5+CD19+ CLL cells in all four patient samples tested, with a mean reduction of 49% at 2000nM after 40 hours *in vitro* incubation (**Figure 2D**, p<0.005). We then addressed whether surface HLA-E downregulation by selinexor was specific to malignant B cells or whether selinexor also modulates expression of HLA-E on lymphocytes from healthy donors. Incubation of selinexor (500-2000nM) for 16 hours with healthy donor PBMC caused a significant downregulation of surface HLA-E expression on normal B cells compared to both normal T cells (p<0.0001) and NK cells p<0.0001) (**Figure 2E**). This data indicates that XPO1 inhibition induces loss of surface HLA-E expression on malignant B cells as well as on normal B cells relative to other lymphocyte populations. Selinexor did not reduce total protein levels of HLA-E in SUDHL4 lymphoma cells (**Figures 2F, G**) and therefore XPO1 mediated downregulation of surface HLA-E was not due to targeted inhibition of HLA-E protein production, but more likely was a result of a reduction in supply of other HLA-E binding substrates that lead to HLA-E upregulation. Indeed, surface expression of HLA-E compared to other HLA molecules is highly sensitive to blockade of newly synthesized protein transport (34, 35) and selinexor potently inhibits protein translation (36).

**Figure 3:**
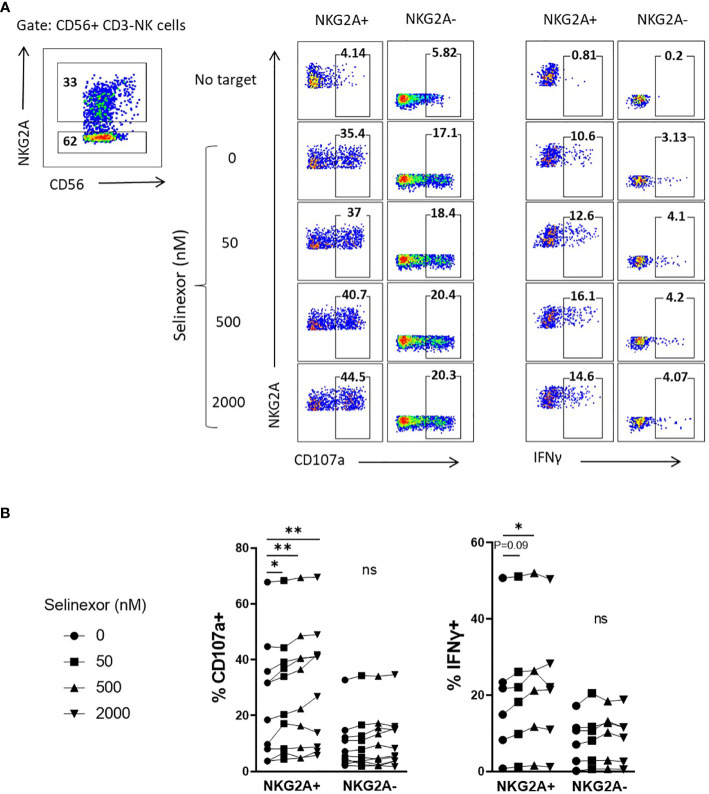
NKG2A+ NK cells are selectively activated by selinexor. **(A, B)** Healthy human PBMC were incubated with IL-15 overnight then co-cultured (E:T 5:1) for 4hrs with SUDHL4 cells pre-treated with selinexor at indicated concentrations or DMSO control for 16hrs. Degranulation (CD107a, n=10) and IFNγ production (n=6) were then assessed on NKG2A+ and NKG2A- CD3-CD56+ NK cells identified as indicated in the representative example A). A representative example of CD107a and IFNγ staining after co-culture of PBMCs with selinexor-treated SUDHL4 is shown in **(A)**. CD107a and IFNγ normalized to the ‘no target’ control is shown in **(B)**. Groups were analysed with repeated measure one-way ANOVA followed by Dunnett’s *post-hoc* test analysis: *P < 0.05; **P < 0.01; ns, not significant.

Taken together these data show that selinexor downregulates HLA-E surface expression on lymphoma cell lines (SUDHL4, JeKo-1 and RAMOS) and primary CLL cells. HLA-E is the ligand for the inhibitory receptor NKG2A and this suggests a potential mechanism by which it may augment NK cell activity (37, 38). We thus hypothesized that selinexor would selectively activate NKG2A+ NK cells against lymphoma cells. Indeed, selinexor induced activation of NKG2A+ and not NKG2A- NK cells against SUDHL4 cells as measured by CD107a expression and IFNγ production (**Figure 3**). As previously described (39), NKG2A+ NK cells compared to NKG2A- NK cells showed enhanced CD107a and IFNγ expression against target cells in the absence of selinexor, however this activation was further enhanced in NKG2A+ cells by the addition of selinexor (**Figure 3**). We noted substantial baseline variability between donors in CD107a and IFNγ expression in the absence of selinexor as previously described (15), however selinexor treatment increased CD107a in all donors tested, and overall IFNγ in three of the six donors tested (**Figure 3B**). These data confirm that selinexor increases NK cell activation against lymphoma cells through downregulation of surface HLA-E, reducing the engagement of this inhibitory receptor and promoting activation of NKG2A+ NK cells.

### XPO1 Inhibition and Not Degradation Is Required for Activation of NKG2A+ NK Cells

**Figure 4:**
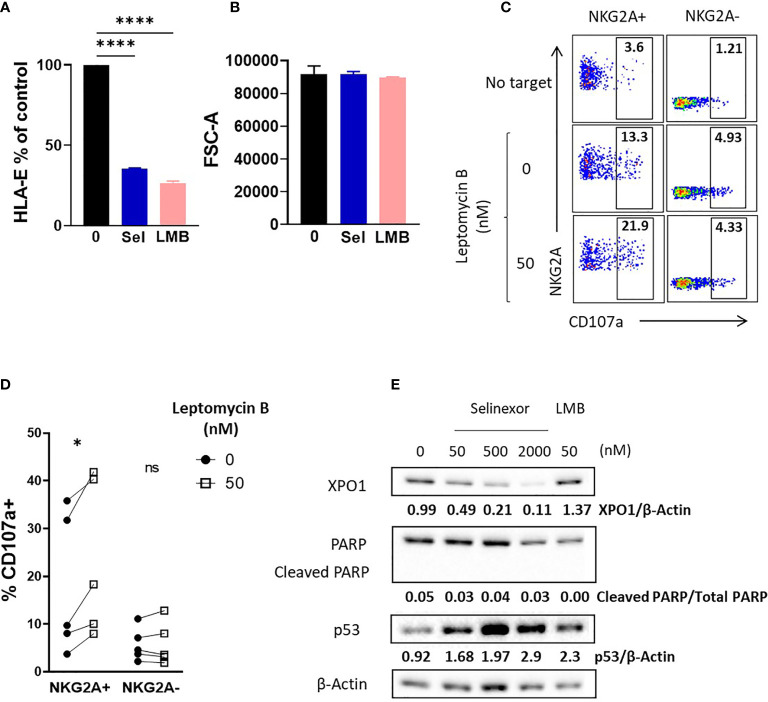
Enhanced NK cell activation is dependent on XPO1 inhibition, and not degradation, in lymphoma cells. **(A, B)** SUDHL4 cells were incubated for 16hrs with leptomycin B (LMB) (50 nM), selinexor ((Sel) 2000 nM) or DMSO control in the presence of the caspase inhibitor QVD then assessed for surface expression of HLA-E (clone 3D12) and cell size as measured by the FSC-A parameter. Summarized data mean ± SEM is shown for HLA-E **(A)** and FSC-A **(B)**. Data was analysed using one-way ANOVA followed by Dunnett’s *post-hoc* test analysis: ****P < 0.0001. **(C, D)** Healthy human PBMC were incubated with IL-15 overnight then co-cultured (E:T 5:1) for 4hrs with SUDHL4 (n=5), cells pre-treated with leptomycin B (50nM) or DMSO control for 16hrs in the presence of the caspase inhibitor QVD. Degranulation (CD107a) was then assessed on NKG2A+ and NKG2A- CD3-CD56+ NK cells identified as indicated in the representative example **(C)**. A representative example of CD107a staining after co-culture of PBMCs with leptomycin B-treated SUDHL4 cells is shown in **(C)**. CD107a normalized to the ‘no target’ control is shown in **(D)**. Groups were analysed with paired t-test: *P < 0.05. **(E)** SUDHL4 cells were incubated for 16hrs with selinexor at indicated concentrations, leptomycin B (LMB, 50 nM) or DMSO control in the presence of the caspase inhibitor QVD. HLA-E, PARP, p53 and β-actin protein levels were then detected by immunoblotting. Representative images are shown from two independent experiments. ns, not significant.

To confirm XPO1 as the target for selinexor induced HLA-E downregulation we utilized an alternative XPO1 inhibitor, leptomycin B. This is a metabolite from Streptomyces which potently inhibits XPO1 function but does not induce XPO1 degradation (40, 41). SUDHL4 cells incubated with leptomycin B (50nM) showed a significant reduction in surface HLA-E expression, mirroring that mediated by selinexor (**Figure 4A**). Decreased HLA-E expression was not caused by changes in cell size as revealed by measurement of forward scatter (**Figure 4B**). In accordance with this, SUDHL4 cells incubated with leptomycin B significantly increased activation of NKG2A+ but not NKG2A- NK cells as measured by CD107a expression (**Figures 4C, D**). We then confirmed using immunoblotting that leptomycin B did not reduce XPO1 protein levels, in contrast to selinexor (**Figure 4E**) as previously reported (40). Leptomycin B did however increase p53 expression, consistent with previous reports (42). In addition, blockade of leptomycin B-induced apoptosis by QVD was confirmed by the absence of cleaved PARP (cPARP) (**Figure 4E**). This data demonstrates that inhibition of XPO1, in the absence of its degradation, is sufficient for HLA-E downregulation on tumor cells and resultant NKG2A+ NK cell activation.

### Selinexor Enhances ADCC in Combination With Anti-CD20 Monoclonal Antibodies

**Figure 5:**
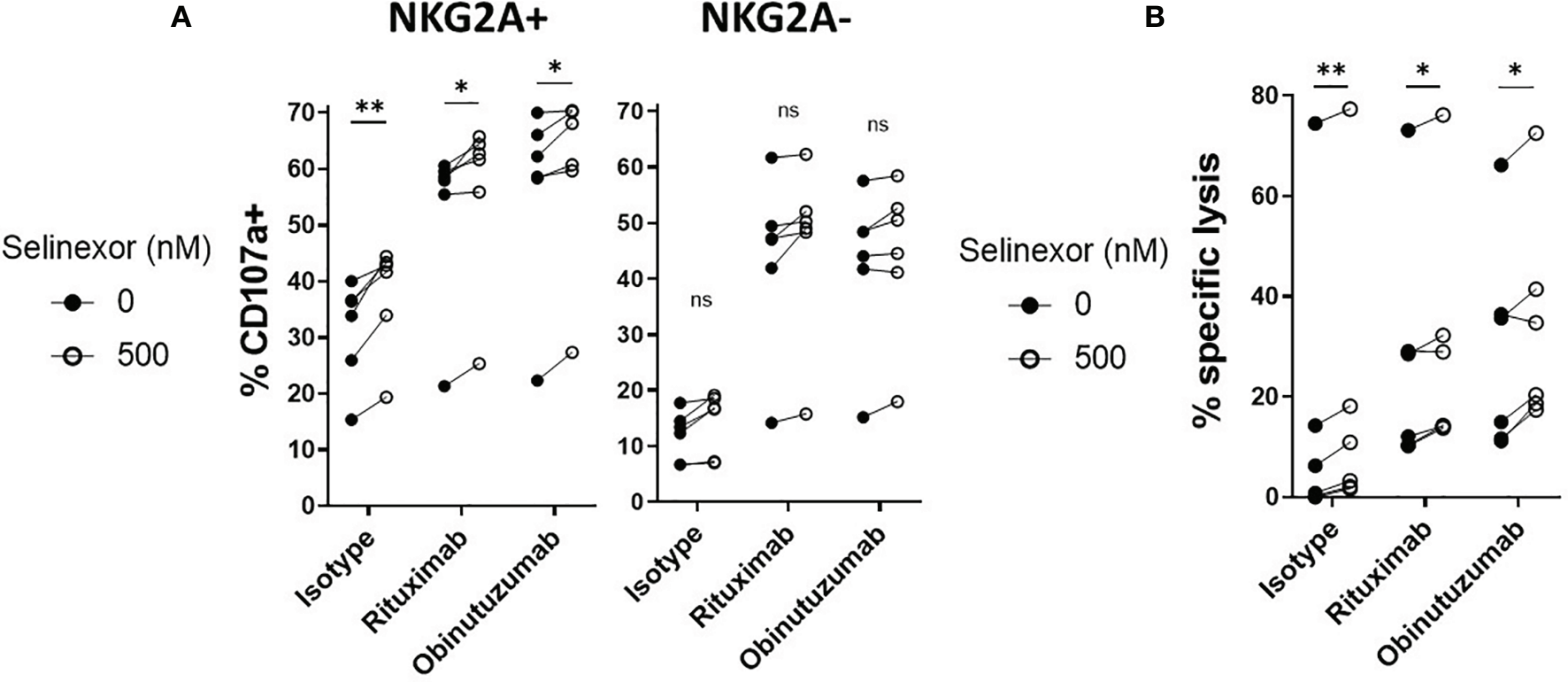
ADCC of NKG2A+ NK cells is enhanced by selinexor. **(A)** Healthy human PBMC were incubated with IL-15 overnight then co-cultured (E:T 5:1) for 4hrs with SUDHL4 cells pre-treated with selinexor (500nM) or DMSO control for 16hrs. 20 minutes prior to co-culture with PBMC, SUDHL4 was incubated with rituximab (rit), obinutuzumab (obz) or isotype control at 1 µg/mL. Degranulation (CD107a) was then assessed on NKG2A+ and NKG2A- CD3-CD56+ NK cells (n=6). CD107a was normalized to the ‘no target’ control and CD107a positivity between selinexor concentrations for each antibody treatment was analysed with paired t-test: *P < 0.05; **P < 0.01. **(B)** Isolated NK cells incubated with IL-15 overnight were co-cultured for 4 hours with SUDHL4 cells (E:T 5:1) that were pre-treated for 16hrs with selinexor (500 nM) or DMSO control in the presence of the caspase inhibitor QVD. 20 minutes prior to co-culture with isolated NK cells, SUDHL4 was incubated with rituximab (rit), obinutuzumab (obz) or isotype control at 1 µg/mL. Cytotoxicity was then assessed using propidium iodide staining on violet dye stained target cells. Data (n=6) was normalized to the corresponding ‘no NK + antibody’ control and analysed with paired t-test: *P < 0.05; **P < 0.01; ns, not significant.

Through their expression of the Fc gamma receptor CD16 (FcgRIIIA), NK cells can elicit anti-tumor functions during anti-CD20 monoclonal antibody (mAbs) treatments in lymphoma (43). As selinexor is currently being clinically evaluated in combination with anti-CD20 antibodies for patients with advanced B cell non-Hodgkin lymphoma (NCT03147885), we addressed whether selinexor could potentiate activity of the anti-CD20 mAbs rituximab and obinutuzumab. SUDHL4 cells were incubated with selinexor (50-2000nM) for 16 hours then cultured with rituximab, obinutuzumab or isotype control for 20 minutes prior to the addition of healthy donor PBMC, containing NK cells. NK cell degranulation in both the NKG2A+ and NKG2A- NK cell populations was increased by the addition of rituximab or obinutuzumab (**Figure 5A**) and in accordance with our previous data, this activation was further increased by selinexor in the NKG2A+ but not NKG2A- NK cell populations (**Figure 5A**). This data indicates that selinexor enhances NK cell activation in the presence of two separate clinically relevant anti-CD20 antibodies. To confirm that this led to increased lysis of lymphoma cells, we performed cytotoxicity assays with isolated NK cells. NK cell specific cytotoxicity against SUDHL4 cells was significantly increased by selinexor (500nM) in the presence of isotype control, rituximab and obinutuzumab (**Figure 5B**). In summary, selinexor enhances ADCC against lymphoma cells in combination with anti-CD20 antibodies.

## Discussion

NK cell dysfunction is a frequent occurrence in human cancers and therapeutic strategies to overcome this are important for sustained tumor regression. Our data identifies that the selective XPO1 inhibitor selinexor disrupts the inhibitory NKG2A:HLA-E axis to activate NK cells against cancer. NKG2A is a novel immune checkpoint target and blocking antibodies against NKG2A are currently in phase 3 clinical trials (15, 38, 44). Selinexor blocks NKG2A mediated inhibitory activity *via* the downregulation of its ligand HLA-E on the surface of tumor cells. This study therefore reveals that selinexor stimulates an anti-cancer response *via* activation of the immune system, in addition to its known direct cytotoxic activity.

NKG2A is an ITIM containing inhibitory receptor expressed by both NK cells and T cells which binds to HLA-E on the surface membrane of target cells (37, 45). Ligation of NKG2A results in recruitment of the tyrosine phosphatase SHP-1 and subsequent inhibition of NK and T cell effector function (15, 46). Downregulation of HLA-E on lymphoma cells by selinexor therefore removes this inhibitory signal and leads to enhanced cytokine production and cytotoxicity of NK cells. We saw no evidence for selinexor induced degradation of HLA-E protein, as total HLA-E protein levels remained unchanged. In contrast, selinexor caused a selective reduction in HLA-E expression at the surface membrane. HLA-E stabilisation at the surface membrane requires constant transport of newly synthesized molecules from the ER, this contrasts with other HLA molecules which are more stable at the surface membrane when transport of new molecules is blocked (34, 35). XPO1 transports ribosomal subunits from the nucleus to the cytoplasm and in accordance with this, selinexor inhibits protein translation selectively in tumor cells with upregulated XPO1 expression (36). Disruption of constant *de novo* protein synthesis has previously been shown to selectively downregulate HLA-E surface expression (34) and this therefore provides a potential mechanism for selective HLA-E downregulation on lymphoma cells by selinexor and leptomycin B. Interestingly, HLA-E has also been shown to be downregulated by bortezomib *via* ER stress in multiple myeloma (34) and by the CDK inhibitor dinaciclib in AML (47).

HLA-E is frequently overexpressed in solid tumors and hematological malignancies including CLL and multiple myeloma (15, 48–50). Therefore selinexor mediated HLA-E downregulation may have broad relevance for activation of NK cells against solid tumors and multiple myeloma, in addition to lymphoma. Indeed, antibody mediated blockade of NKG2A:HLA-E interactions has been shown to activate NK cells *in vitro* and *in vivo* against both lymphoma and solid tumor cells (15) and the blocking anti-NKG2A antibody monolizumab is currently in phase 3 clinical trials for Head and Neck cancer in combination with the anti-EGFR antibody cetuximab (NCT04590963). In addition, NKG2A blockade acted in concert with anti-PD-1 antibodies to enhance tumor regression and promoted ADCC in combination with cetuximab (15). These combination strategies highlight the utility of the results from this study showing that selinexor enhanced ADCC against lymphoma cells coated with obinutuzumab and rituximab. Selinexor is currently being assessed in a clinical trial in combination with anti-CD20 antibodies for patients with advanced B cell non-Hodgkin lymphoma (NCT03147885) and HLA-E downregulation by selinexor may therefore contribute to ADCC in this setting. A recent study identified that HLA-E suppresses NK cell sensitivity to tumor cells and that resistance to immune checkpoint blockade therapy in multiple clinical studies correlates with an NK sensitivity gene signature, including HLA-E (51). This data indicates that therapies which can stimulate NK activation, for example by disruption of NKG2A:HLA-E interactions, may overcome immune checkpoint blockade resistance. Selinexor is currently in clinical trials in combination with anti-CTLA-4 and anti-PD-1 antibodies (NCT04850755) and it would therefore be of interest to assess the contribution of NK cells to efficacy in this setting. Importantly, the concentration of selinexor required to downregulate HLA-E is achievable in patients during selinexor treatment, with a Cmax of 1-2µM in patient plasma reported during clinical trials (52).

Because XPO1 is upregulated in tumors and selinexor selectively blocks translation in tumor cells (36), a potential advantage for blocking NKG2A:HLA-E interactions *via* selinexor rather than antibodies is that selinexor may retain expression of HLA-E on non-tumor cells. This is important because NKG2A:HLA-E interactions are crucial for NK cell education and the prevention of lysis of healthy cells (53). Selinexor downregulated HLA-E expression on both malignant and normal B cells to a greater extent than on normal T cells or NK cells. In addition, auto-immune side-effects are not seen in patients treated with selinexor (54), indicating that selinexor does not promote NK cell directed killing of non-malignant cells.

In murine models, selinexor increased the frequency of both T cells and NK cells (55) however the mechanism for this is unknown. This data implies however that XPO1 inhibition may have a dual role in promotion of NK activity *via* sensitizing tumors to NK mediated destruction and simultaneously increasing NK cell frequency. Furthermore, we found that selinexor increased IFNγ production by NK cells. This is important because IFNγ has key roles in the promotion of adaptive immunity (7), stimulation of macrophage activation and antibody-dependent cellular phagocytosis (ADCP) (56), as well as in the suppression of liver metastases (7, 8). Importantly, XPO1 inhibition did not significantly affect human NK cell mediated ADCC, direct cytotoxicity or viability (57). In addition, selinexor increased NK cell frequency in a murine tumor model *in vivo* (55). Together, this indicates that the findings from this study may be relevant *in vivo*, where both target cells and NK cells will simultaneously be exposed to selinexor. In addition to NK cells, inhibition of NKG2A has also been shown to enhance the efficacy of cancer vaccines in murine tumor models *via* CD8+ T cell activation (58) and therefore selinexor mediated HLA-E downregulation may also promote T cell activity. Interestingly, previous studies have revealed that selinexor pre-treatment increase T cell activation against B cell malignancies (28) and breast cancer cells (27) however the contribution of HLA-E/NKG2A interactions in these settings were not investigated. Based on our data, it is plausible that this may have been due to disruption of the NKG2A:HLA-E axis by selinexor.

In addition to its role in tumors, XPO1 is also crucial for COVID-19 infection, with selinexor recently shown to inhibit COVID-19 mediated pathology and neutrophilic rhinitis (59). The contribution of NKG2A:HLA-E interactions in this model was not investigated however it is interesting to note that inhibition of NKG2A increases IFNγ mediated suppression of neutrophils (60). Antibody mediated blockade of NKG2A has been proposed as a novel COVID-19 treatment (61) and therefore selinexor mediated downregulation of HLA-E may also potentially participate in the anti-viral efficacy of selinexor.

In conclusion, we identify a novel anti-tumor mechanism for XPO1 inhibitors *via* HLA-E downregulation and resultant activation of NKG2A+ NK cells. This data indicates that NK cells may contribute to the therapeutic efficacy of selinexor and that selinexor may synergize with NK cell targeted therapies for the treatment of patients with cancer. Whether NK cells are associated with patient outcome following selinexor treatment is currently under investigation.

## Data Availability Statement

The raw data supporting the conclusions of this article will be made available by the authors, without undue reservation.

## Ethics Statement

The studies involving human participants were reviewed and approved by National Research Ethics Committee and Institutional Review Boards at the University of Southampton. The patients/participants provided their written informed consent to participate in this study.

## Author Contributions

MB, SK, and YL conceived and designed the study. JF, AD, SK, and MB collected and analysed the data. MB, SK, and JF wrote the original draft. JF, CW, PJ, FF, MC, YL, SK, and MB reviewed and edited the manuscript. All authors contributed to the article and approved the submitted version.

## Funding

Research reported in this article was supported by funding from Leukaemia UK (John Goldman Fellowship) and Karyopharm Therapeutics to MB and from the MRC (DTP award MR/N014308/1 and 519241101).

## Conflict of Interest

CW and YL are employees and stockholders of Karyopharm Therapeutics. MB received research funding from Karyopharm Therapeutics. MC is a retained consultant for BioInvent International and has performed educational and advisory roles for Roche, Boehringer Ingelheim, Baxalta, Merck KGaA and GLG. He has received research funding from Bioinvent, Roche, Gilead, iTeos, UCB and GSK.

The authors declare that this study received funding from Karyopharm Therapeutics. The funder initiated contact with MB and SK and reviewed the manuscript prior to publication.

The remaining authors declare that the research was conducted in the absence of any commercial or financial relationships that could be construed as a potential conflict of interest.

## Publisher’s Note

All claims expressed in this article are solely those of the authors and do not necessarily represent those of their affiliated organizations, or those of the publisher, the editors and the reviewers. Any product that may be evaluated in this article, or claim that may be made by its manufacturer, is not guaranteed or endorsed by the publisher.
